
JOURNAL INFORMATION
==============================
NLM Title Abbreviation: Hum Brain Mapp
Journal ID: Hum Brain Mapp
NLM Journal ID: 9419065
Journal ID: HBM

Human Brain Mapping

ISSN: 1065-9471
EISSN: 1097-0193
Publisher: Wiley

ARTICLE INFORMATION
==============================
PMCID: PMC11474658
DOI: 10.1002/hbm.25936
Article ID: HBM25936
Article version: 1
Subjects: Issue Information, Issue Information

Editorial board ‐ TOC

Electronic publication date: 2023 Sep 15
Publication date: 2023 Oct 15
Volume: 44
Issue: 15
First page: 4969
Last page: 4971
Copyright: © 2023 The Authors. Human Brain Mapping published by Wiley Periodicals LLC.
License: This is an open access article under the terms of the http://creativecommons.org/licenses/by/4.0/ License, which permits use, distribution and reproduction in any medium, provided the original work is properly cited.
License URL: https://creativecommons.org/licenses/by/4.0/

Figure count: 0
Table count: 0
Page count: 7

source-schema-version-number: 2.0
cover-date: 15 October, 2023
details-of-publishers-convertor: Converter:WILEY_ML3GV2_TO_JATSPMC version:6.4.9 mode:remove_FC converted:15.10.2024

==============================

### HUMAN BRAIN MAPPING

Open Access

Volume 44, Issue 15, 15 October, 2023

View this journal online at wileyonlinelibrary.com

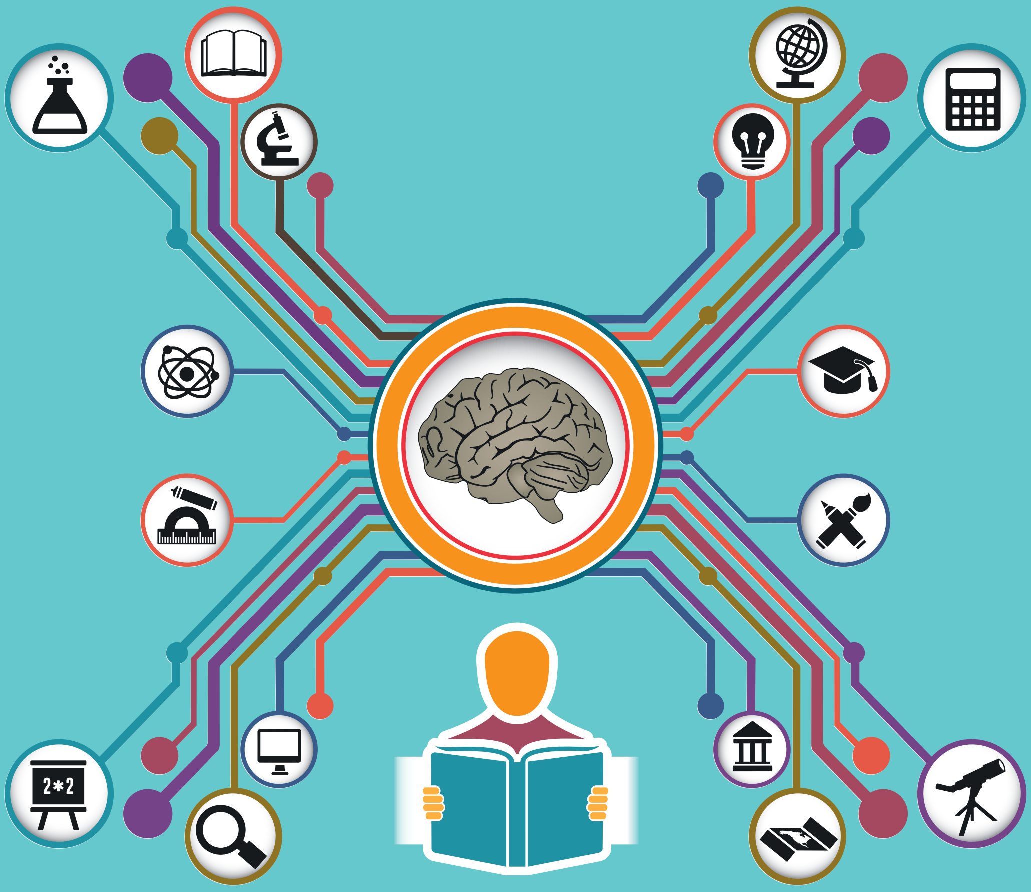

WILEY

ISSN 1097-0193

### HUMAN BRAIN MAPPING

### A Journal Devoted to Functional Neuroanatomy and Neuroimaging

### *Editor-in-Chief*

**Simon B. Eickhoff**
Institute for Systems Neuroscience
Heinrich-Heine University, Düsseldorf, Germany

### *Deputy Editors-in-Chief*

**Gary Egan**
Monash Biomedical Imaging
Monash University, Victoria, Australia

**Sophia Frangou**
Icahn School of Medicine
at Mount Sinai, New York, USA

**Martijn van den Heuvel**
Brain Center Rudolf Magnus,
University of Medical Center,
Utrecht, Netherlands

### *Associate Editors*

**Annabel Chen**
Nanyang Technological University, Singapore

**Michael J. Farrell**
Monash University, Victoria, Australia

**Massimo Filippi**
Università Vita-Salute San Raffaele, Milan,
Italy

**Sophia Frangou**
Icahn School of Medicine at Mount Sinai
New York, USA

**Cheryl L. Grady**
Rotman Research Institute
Toronto, Canada

**Joachim Gross**
Centre for Cognitive Neuroimaging
University of Glasgow, Glasgow, UK

**Martin A. Lindquist**
John Hopkins, Bloomberg School of Public
Health, MD, USA

**Tomáš Paus**
Rotman Research Institute
Toronto, Canada

**Indira Tendolkar**
Radboud University, Nijmegen,
The Netherlands

**Paul Thompson**
Laboratory of Neuro Imaging,
University of Southern California,
Los Angeles, CA, USA

**Helen Zhou**
Yong Loo Lin School of Medicine, National
University of Singapore, Singapore

### *Editorial Board*

**Susan Bookheimer**
Center for Cognitive Neuroscience
UCLA School of Medicine, USA

**Allen R. Braun**
NIDCD, National Institutes of Health
Bethesda, USA

**Christian Buechel**
Univ-Krankenhaus Eppendorf
Hamburg, Germany

**Marco Catani**
King's College London, UK

**Franco Cauda**
University of Turin, Italy

**Vincent Clark**
University of New Mexico, USA

**Olivier David**
Université Joseph Fourier, France

**Mark D'Esposito**
University of California, Berkeley,
USA

**Julien Doyon**
Functional Neuroimaging Unit
University of Montreal, Canada

**Eugene Duff**
University of Oxford, UK

**Timothy Q. Duong**
Research Imaging Institute,
University of Texas Health Science
Center, USA

**Paul Fletcher**
University of Cambridge, UK

**Jia-Hong Gao**
Brain Research Imaging
Center The University of Chicago,
USA

**David C. Glahn**
Yale University School of Medicine
Hartford, USA

**Gregory S. Hickok**
Center for Cognitive
Neuroscience University of California,
Irvine, USA

**Barry Horwitz**
NIDCD, National Institutes of Health
USA

**Peter Jezzard**
John Radcliffe Hospital
University of Oxford, UK

**David N. Kennedy**
Harvard Medical School, USA

**Neda Ladbon-Bernasconi**
Montreal Neurological Institute
Canada

**Nicholas Lange**
Harvard Medical School, USA

**Tatia M. C. Lee**
State Key Laboratory of Brain &
Cognitive Sciences, The University of
Hong Kong

**Louis Lemieux**
UCL Institute of Neurology, UK

**Bernard Mazoyer**
CNRS/CEA, Université de Bordeaux
France

**Gregory McCarthy**
Yale University, USA

**A. Randy McIntosh**
Rotman Research Institute
Toronto, Canada

**Janine Mendola**
McGill Vision Research
McGill University, Canada

**Thomas Nichols**
University of Warwick, UK

**William Penny**
Wellcome Trust Centre for
Neuroimaging, UCL Institute of
Neurology, UK

**Laurent Petit**
CNRS, Université de Bordeaux,
France

**Russel A. Poldrack**
Stanford Unversity, USA

**Jean-Baptiste Poline**
Imagen Orsay Service Hospitalier
Frédéric Joliot, France

**Stephen Smith**
Center for Functional MRI of the
Brain, University of Oxford, UK

**Elizabeth Sowell**
Children'sHospital Los Angeles, USA

**Li Hai Tan**
Shenzhen Institute of Neuroscience
Shenzhen, P.R. China

**Kate Watkins**
John Radcliffe Hospital
University of Oxford, UK

**Richard G. Wise**
Cardiff University, UK

**Roger Woods**
UCLA School of Medicine, USA

**Mark Woolrich**
Oxford Centre for Human Brain
Activity, University of Oxford, UK

**Jinhu Xiong**
University of Iowa, USA

**Robert Zatorre**
MontrealNeurological Institute, Canada

**Open Access and Copyright**
All articles published by *Human Brain Mapping* submitted after January 1, 2020 are fully open access: immediately freely available to read, download, and share. All articles accepted from August 6, 2019 are published under the terms of a Creative Commons license.
Copyright on any research article published by *Human Brain Mapping* is retained by the author(s).

Further information about open access licenses and copyright can be found at: https://authorservices.wiley.com/open-research/open-access/index.html. Information on the licenses used by *Human Brain Mapping* can be found at: https://onlinelibrary.wiley.com/journal/10970193.

**Disclaimer**
The Publisher and Editors cannot be held responsible for errors or any consequences arising from the use of information contained in this journal; the views and opinions expressed do not necessarily reflect those of the Publisher and Editors, neither does the publication of advertisements constitute any endorsement by the Publisher and Editors of the products advertised. Wiley Open Access articles posted to repositories or websites are without warranty from Wiley of any kind, either express or implied, including, but not limited to, warranties of merchantability, fitness for a particular purpose, or non-infringement. To the fullest extent permitted by law Wiley disclaims all liability for any loss or damage arising out of, or in connection, with the use of or inability to use the content.

### HUMAN BRAIN MAPPING

A Journal Devoted to
Functional Neuroanatomy
and Neuroimaging

VOLUME 44, NUMBER 15, 15 OCTOBER 2023

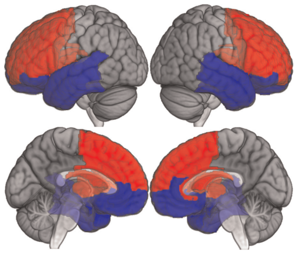

*See article by, Sokołowski et al, pages 5013-5029, this issue*

### CONTENTS

◆ *4972* Divergent auditory activation in relation to inhibition task performance in children and adults
**Sam van Bijnen, Joona Muotka, and Tiina Parviainen**
***Published online 26 July 2023***

◆ *4986* Data-driven electrical conductivity brain imaging using 3 T MRI
**Kyu-Jin Jung, Stefano Mandija, Chuanjiang Cui, Jun-Hyeong Kim, Mohammed A. Al-masni, Thierry G. Meerbothe, Mina Park, Cornelis A. T. van den Berg, and Dong-Hyun Kim**
***Published online 19 July 2023***

◆ *5002* Functional changes in fusional vergence-related brain areas and correlation with clinical features in intermittent exotropia using functional magnetic resonance imaging
**Weijia Zhang, Nanxi Fei, Yachen Wang, Bingbing Yang, Zhihan Liu, Luyao Cheng, Junfa Li, Junfang Xian, and Tao Fu**
***Published online 4 August 2023***

◆ *5013* Neuropsychiatric symptoms and imbalance of atrophy in behavioral variant frontotemporal dementia
**Andrzej Sokołowski, Ashlin R. K. Roy, Sheng-Yang M. Goh, Emily G. Hardy, Samir Datta, Yann Cobigo, Jesse A. Brown, Salvatore Spina, Lea Grinberg, Joel Kramer, Katherine P. Rankin, William W. Seeley, Virginia E. Sturm, Howard J. Rosen, Bruce L. Miller, and David C. Perry**
***Published online 20 July 2023***

◆ *5030* Planning ahead: Predictable switching recruits task-active and resting-state networks
**Danielle L. Kurtin, Garazi Araña-Oiarbide, Romy Lorenz, Ines R. Violante, and Adam Hampshire**
***Published online 20 July 2023***

(*continued on overleaf*)

(*continued from overleaf*)

◆ *5047* Quantitative susceptibility mapping identifies hippocampal and other subcortical grey matter tissue composition changes in temporal lobe epilepsy
**Oliver C. Kiersnowski, Gavin P. Winston, Lorenzo Caciagli, Emma Biondetti, Maha Elbadri, Sarah Buck, John S. Duncan, John S. Thornton, Karin Shmueli, and Sjoerd B. Vos**
***Published online 26 July 2023***

◆ *5065* Proactive and reactive language control in bilingual language production revealed by decoding sustained potentials and electroencephalography oscillations
**Zhaoqi Zhang, Fengyang Ma, and Taomei Guo**
***Published online 28 July 2023***

◆ *5079* The functional connectome of 3,4-methyldioxymethamphetamine-related declarative memory impairments
**Rebecca C. Coray, Josua Zimmermann, Amelie Haugg, Markus R. Baumgartner, Andrea E. Steuer, Erich Seifritz, Ann-Kathrin Stock, Christian Beste, David M. Cole, and Boris B. Quednow**
***Published online 2 August 2023***

◆ *5095* Improved dynamic distortion correction for fMRI using single-echo EPI and a readout-reversed first image (REFILL)
**Simon Daniel Robinson, Beata Bachrata, Korbinian Eckstein, Saskia Bollmann, Steffen Bollmann, Shota Hodono, Martijn Cloos, Monique Tourell, Jin Jin, Kieran O'Brien, David C. Reutens, Siegfried Trattnig, Christian Enzinger, and Markus Barth**
***Published online 7 August 2023***

◆ *5113* Multishell diffusion MRI reveals whole-brain white matter changes in HIV
**Silvia Minosse, Eliseo Picchi, Allegra Conti, Francesca di Giuliano, Francesco di Ciò, Loredana Sarmati, Elisabetta Teti, Silvia de Santis, Massimo Andreoni, Roberto Floris, Maria Guerrisi, Francesco Garaci, and Nicola Toschi**
***Published online 30 August 2023***

◆ *5125* Indirect evidence for altered dopaminergic neurotransmission in very premature-born adults
**David Schinz, Benita Schmitz-Koep, Juliana Zimmermann, Elin Brandes, Marlene Tahedl, Aurore Menegaux, Juergen Dukart, Claus Zimmer, Dieter Wolke, Marcel Daamen, Henning Boecker, Peter Bartmann, Christian Sorg, and Dennis M. Hedderich**
***Published online 22 August 2023***

(*continued on overleaf*)

(*continued from overleaf*)

◆ *5139* MRAβ: A multimodal MRI-derived amyloid-β biomarker for Alzheimer's disease
**Yu Zhang, Xi Li, Yi Ji, Hao Ding, Xinjun Suo, Xiaoxi He, Yingying Xie, Meng Liang, Shijie Zhang, Chunshui Yu, and Wen Qin**
***Published online 14 August 2023***

◆ *5153* Robust hierarchically organized whole-brain patterns of dysconnectivity in schizophrenia spectrum disorders observed after personalized intrinsic network topography
**Erin W. Dickie, Saba Shahab, Colin Hawco, Dayton Miranda, Gabrielle Herman, Miklos Argyelan, Jie Lisa Ji, Jerrold Jeyachandra, Alan Anticevic, Anil K. Malhotra, and Aristotle N. Voineskos**
***Published online 22 August 2023***

◆ *5167* Multimodal fusion of multiple rest fMRI networks and MRI gray matter via parallel multilink joint ICA reveals highly significant function/structure coupling in Alzheimer's disease
**K. M. Ibrahim Khalilullah, Oktay Agcaoglu, Jing Sui, Tülay Adali, Marlena Duda, and Vince D. Calhoun**
***Published online 22 August 2023***

◆ *5180* MR-based spatiotemporal anisotropic atrophy evaluation of hippocampus in Alzheimer's disease progression by multiscale skeletal representation
**Na Gao, Zhiyuan Liu, Yuesheng Deng, Hantao Chen, Chenfei Ye, Qi Yang, and Ting Ma**
***Published online 22 August 2023***
